# Direct evidence of two interatomic relaxation mechanisms in argon dimers ionized by electron impact

**DOI:** 10.1038/ncomms11093

**Published:** 2016-03-22

**Authors:** Xueguang Ren, Elias Jabbour Al Maalouf, Alexander Dorn, Stephan Denifl

**Affiliations:** 1 Max-Planck-Institut für Kernphysik, Saupfercheckweg 1, 69117 Heidelberg, Germany; 2 Physikalisch-Technische Bundesanstalt, Bundesallee 100, 38116 Braunschweig, Germany; 3 Institut für Ionenphysik und Angewandte Physik, Universität Innsbruck, Technikerstrasse 25, 6020 Innsbruck, Austria

## Abstract

In weakly bound systems like liquids and clusters electronically excited states can relax in inter-particle reactions via the interplay of electronic and nuclear dynamics. Here we report on the identification of two prominent examples, interatomic Coulombic decay (ICD) and radiative charge transfer (RCT), which are induced in argon dimers by electron collisions. After initial ionization of one dimer constituent ICD and RCT lead to the ionization of its neighbour either by energy transfer to or by electron transfer from the neighbour, respectively. By full quintuple-coincidence measurements, we unambiguously identify ICD and RCT, and trace the relaxation dynamics as function of the collisional excited state energies. Such interatomic processes multiply the number of electrons and shift their energies down to the critical 1–10 eV range, which can efficiently cause chemical degradation of biomolecules. Therefore, the observed relaxation channels might contribute to cause efficient radiation damage in biological systems.

## Abstract (web summary)

Inter-particle reactions in weakly bound systems are often difficult to pinpoint by detecting exclusively the kinetic energy of the produced ions. Here the authors present a full-coincidence experiment, where inter-particle reaction channels are determined by the measurement of all outgoing particles.

## Graphical abstract

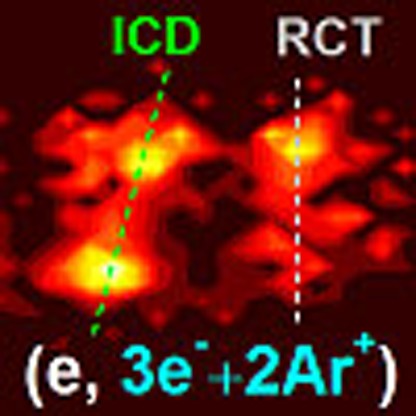

One of the most fundamental questions in physics, chemistry and biology is the way of relaxation of atomic and molecular systems after initial (electronic) excitation. This relaxation results from the behaviour of nature to reach the lowest energetic state. For the transition from isolated atoms and molecules in the gas phase to the weakly bound systems (van der Waals clusters and liquids), a wealth of new energy transfer phenomena emerges like the recently discovered interatomic Coulombic decay (ICD), which is enabled only by the presence of the environment^1^,^2^. In ICD, first predicted by Cederbaum *et al.*^3^ on theoretical grounds, an ionized or excited species relaxes by transferring its excess energy to the neighbour, where it leads to ejection of a low-energy electron and two repulsive ions at a distance of few Ångströms (Coulomb explosion).

Experimentally ICD was confirmed for the first time in 2003 (ref. 4). From that time onwards many experimental studies have been carried out using light sources such as synchrotron radiation^5^ or free electron lasers^6^. In contrast, ICD studies involving ionization or excitation induced in clusters by particles like ions^7^,^8^,^9^ or electrons are scarce^10^,^11^,^12^ though, in principle, electron impact offers the advantage of populating optically forbidden states. However, experimentally one must face a major problem: in the outgoing scattering channel three electrons are present, two ejected ones and the scattered one, which lost an unknown fraction of its energy due to the excitation event. In addition two energetic ions are produced.

All previous experiments based on electron impact relied on the detection of two ions formed and on the determination of the kinetic energy release (KER; representing the sum of kinetic energies of the fragment ions formed in the outgoing channel). However, in this case the assignment to ICD is not ambiguous, since other competitive but fundamental relaxation mechanisms like radiative charge transfer (RCT)^13^,^14^ may occur. In the latter case, one atom in the dimer becomes doubly ionized and subsequently an electron is transferred from the neutral atom, which is accompanied by release of a photon. Since in this case also two fragment ions are formed, which repel each other, the final observable (the KER) is very similar to the one for an ICD process.

In the present electron ionization study of argon dimers we have solved this problem by measuring all five charged particles in the outgoing reaction channel, that is, detecting both resulting ions and all three electrons. This full-coincidence experiment allows an unambiguous identification of the relaxation process accompanied by the electron ionization process. We show that both relaxation mechanisms are present and elucidate the ratio between both mechanisms. Moreover, we find a markedly enhanced production of low-energy electrons in the relaxation of argon dimer. The results suggest that the observed decay processes may have a significant influence on the radiation damage of biological tissues like DNA^15^,^16^.

## Results

### KER of fragment ions

**Figure 1:**
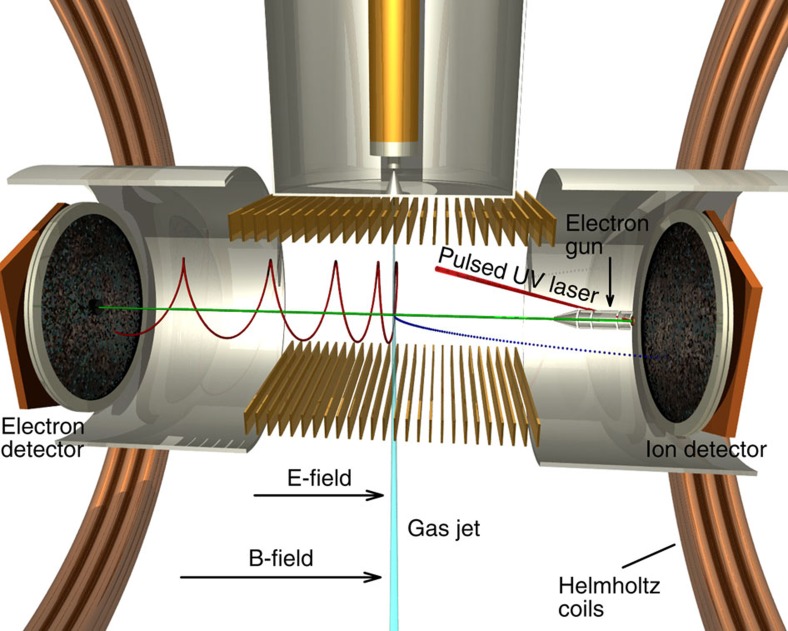
Experimental set-up. Schematic view of the reaction microscope used to perform electron-scattering experiments on argon dimers. The pulsed projectile electron beam (horizontal green line) is crossing an argon supersonic gas jet (vertical light blue line). The projectiles are produced by photoemission from a tantalum photocathode illuminated by a pulsed ultraviolet (UV) laser beam (red conic line). The electrons and ions emerging from a collision in the reaction volume are extracted by means of a homogeneous electric field (E-field) created by a series of parallel electrodes and detected by two facing microchannel plate detectors. A pair of Helmholtz coils generates an uniform magnetic field (B-field), which forces the electrons to move along cyclotron trajectories (exemplary red spiral line), and thus confines their motion in transversal extension.

**Figure 2:**
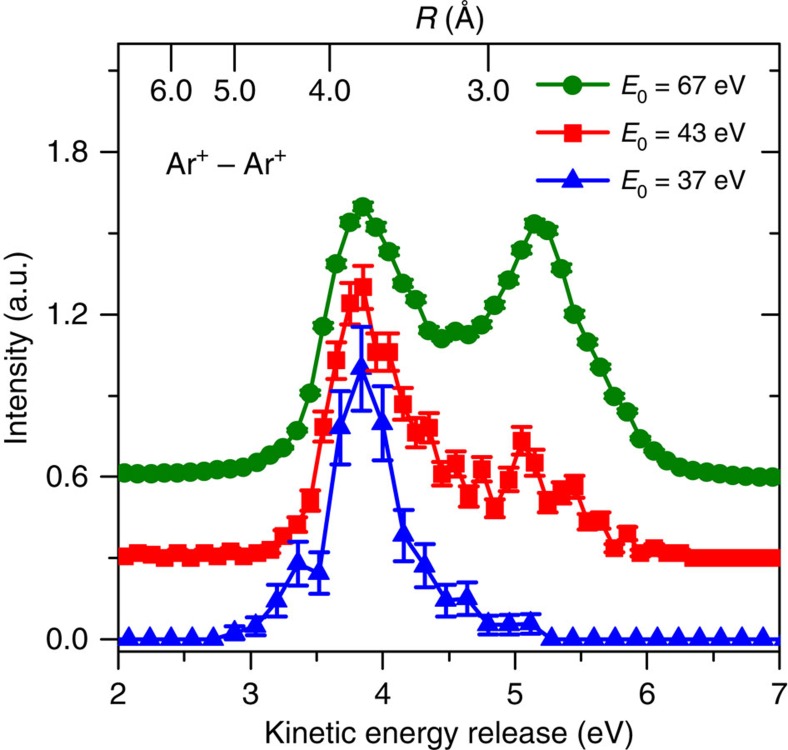
Measured kinetic energy of Ar^+^ ion pairs. KER spectra for the production of two repulsive Ar^+^ ions after break-up of the dimer at projectile electron energies of *E*_0_=37 (blue triangles), 43 (red squares) and 67 eV (green circles). The spectra are normalized at the 3.8-eV peak, and for better visibility the data for *E*_0_=43 and 67 eV are shifted upwards by 0.3 and 0.6 a.u. (arbitrary units of signal intensity), respectively. The top scale shows the internuclear distance *R* at the instant when Coulomb explosion starts, that is, when the second Ar atom is ionized. *R* is determined from the associated Coulomb repulsion potential, which in good approximation corresponds to the observed KER. The error bars of the data points are defined as s.d. and are calculated as square root of the experimental count number.

**Figure 3:**
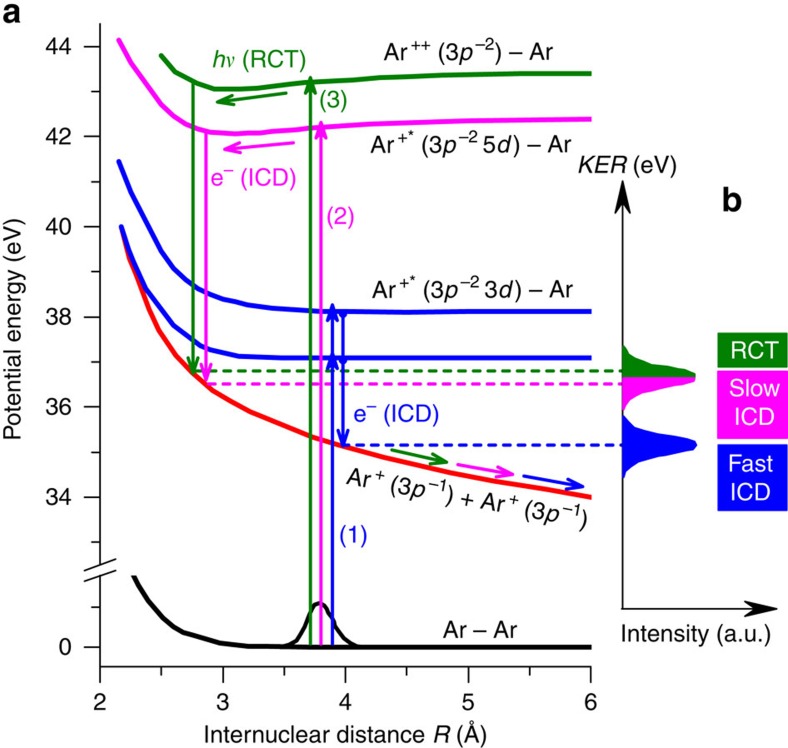
Schematic view of potential energy curves and transitions. (**a**) Illustration of the three interatomic relaxation processes following ionizing collisions of electrons and argon dimers. In the fast ICD ((1) and blue arrows) low-lying satellite states are populated. The lifetime of these states with respect to ICD decay to the repulsive Ar^+^−Ar^+^ final state is short. Thus, no vibrational motion to smaller internuclear distance is possible and the resulting KER is relatively low. For the slow ICD process ((2) and magenta arrows) higher-lying satellite states are populated, which have longer lifetime and allow vibrational motion. Here ICD occurs at shorter internuclear distance resulting in higher KER. For the process of RCT ((3) and green arrows), one dimer atom is doubly ionized (Ar^++^−Ar). The dimer decays to the Ar^+^−Ar^+^ state by transferring an electron from the neighbour atom to Ar^++^ accompanied by photon emission. This process preferentially takes place at shorter *R* and, thus, also contributes to the higher KER peak. The potential energy curves shown are adapted from refs 18, 20. (**b**) The expected relative KER peak positions for the three different processes.

A multi-particle coincidence spectrometer (reaction microscope) designed for electron impact experiments^10^,^17^ (Fig. 1) is employed to measure the momentum vectors and, consequently, the kinetic energies for all the final-state electrons and fragment ions. Figure 2 shows the KER spectra for Ar^+^+Ar^+^ fragment ions measured at increasing projectile energies of *E*_0_=37, 43 and 67 eV. For the highest impact energy (67 eV) the overall shape of the ion yield showing two peaks at 3.8 and 5.2 eV is in good agreement with theoretical predictions^18^ and previous experimental studies determining the KER^10^,^19^. In a previous electron impact study on argon dimers Yan *et al.*^11^ ascribed the peak at lower KERs to ICD and the other peak to RCT. This assignment was based on a simple comparison of the possible timescales of these processes since no information on the electrons was collected in the experiment. However, a recent theoretical study by Miteva *et al.*^18^ has drawn a different picture suggesting that ICD should also contribute to the higher KER peak. Possible ionization and relaxation mechanisms are schematically shown in Fig. 3. They are the following: (1) fast ICD, for which vertical Franck–Condon transitions populate low-lying satellite states of the form (Ar^+*^(3*p*^−2^ *nl*)−Ar) with shallow potential minima (between 36 and 130 meV binding energy) close to the equilibrium internuclear distance (*R*_eq_) of the neutral argon dimer at 3.8 Å. They will quickly decay at *R*_eq_ via ICD to the repulsive Ar^+^−Ar^+^ final state due to the short lifetime (∼100 fs) compared with the vibrational periods (∼1 ps) and form the low-KER peak at 3.8 eV; (2) slow ICD, where higher ionization satellites are populated, which have deeper potential wells (250–340 meV) being shifted to shorter *R* around 3 Å (ref. 18). These have one order of magnitude shorter vibrational periods (∼200 fs) than their ICD lifetimes^18^. Hence vibrational motion can occur, resulting in ICD preferentially occurring at shorter *R* forming the higher KER peak; (3) RCT for population of the states (Ar^++^−Ar) above the double ionization threshold of the argon atom (43.4 eV) (ref 20). The system can decay to the Ar^+^−Ar^+^ state by transferring an electron from the neighbour atom to Ar^++^ accompanied by photon emission. Also this radiative process is rather slow (approximately nanosecond regime)^13^ enabling vibrational motion, and it preferentially takes place at shorter *R*, and thus contributes to the higher KER peak.

Our observation of only one peak at 3.8 eV KER for *E*_0_=37 eV in Fig. 2, where only the lowest states are reached, confirms the interpretation that these states decay fast and lead to low KER. For increasing impact energy 43 eV a second peak at higher KER emerges due to the population of the higher-lying states. We purely attribute the yield to the slow ICD process since the RCT channel is absent at this energy. The projectile energy of 67 eV is sufficient for double ionization of one argon atom and, therefore, to open the RCT channel in addition to the ICD channels. The KER spectra show increasing intensity at 5.2 eV, which might be due to both RCT and the increased cross-sections for the slow ICD process.

### Two-dimensional energy correlation map

**Figure 4:**
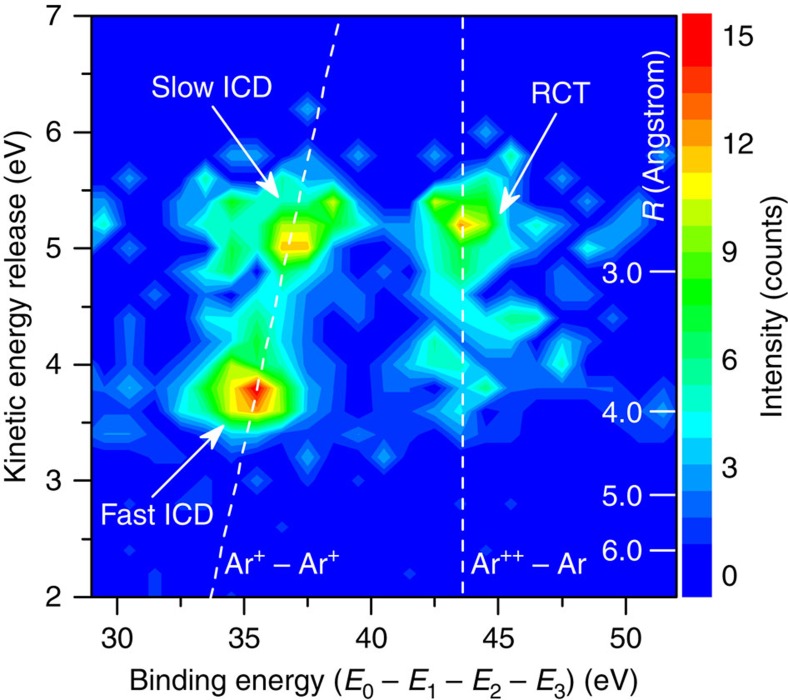
Quintuple-coincidence data (2 ions+3 electrons) for *E*_0_=67 eV. Correlation map between binding energy (*E*_B_) and KER. The horizontal axis shows *E*_B_, the difference of incident electron energy and the sum energy of all the three outgoing electrons. The *y* axis shows the KER of the two Ar^+^ ions. Regions ascribed to ICD and RCT are indicated. The right scale shows the internuclear distance *R*.

To clearly identify and separate the underlying relaxation processes above the threshold of both processes we carried out the fully quintuple-coincidence experiment of argon dimer at *E*_0_=67 eV. From this we obtain a two-dimensional correlation map between the KER and the binding energy (*E*_B_), as shown in Fig. 4. Here we define *E*_B_ as the initial projectile energy *E*_0_ minus the sum energy of all three outgoing electrons (*E*_B_=*E*_0_−(*E*_1_+*E*_2_+*E*_3_)). *E*_B_ is the energy of the state reached after both initially bound electrons are ejected, that is, for ICD the Ar^+^−Ar^+^ repulsive states and for RCT the intermediate state Ar^++^−Ar (Fig. 3). Accordingly, in the two-dimensional map we identify the peak at about *E*_B_=35 eV and 3.8 eV KER to the fast ICD process and the peak at about *E*_B_=37 eV and 5.2 eV KER to the slow ICD process. Also the linear relation *E*_B_=2·IP_Ar_+KER between *E*_B_, that is, the point reached at the repulsive Ar^+^−Ar^+^ state and KER indicated by the dashed line with slope 1 is seen in the data (IP_Ar_: Ar ionization energy). In contrast, the region at higher *E*_B_ is characterized by transitions to doubly ionized states coupled with a neutral argon atom Ar^++^−Ar, that is, these are precursor states that will lead via RCT to the Ar^+^−Ar^+^ state. The additional *E*_B_ observed here is taken by the emitted photon. This result shows clearly that the RCT relaxation mechanism is present in the clusters once the energy of the electron is above the threshold since energetically only the Ar^+^−Ar^+^ ion ground state channel is open.

### ICD electron energy spectra

**Figure 5:**
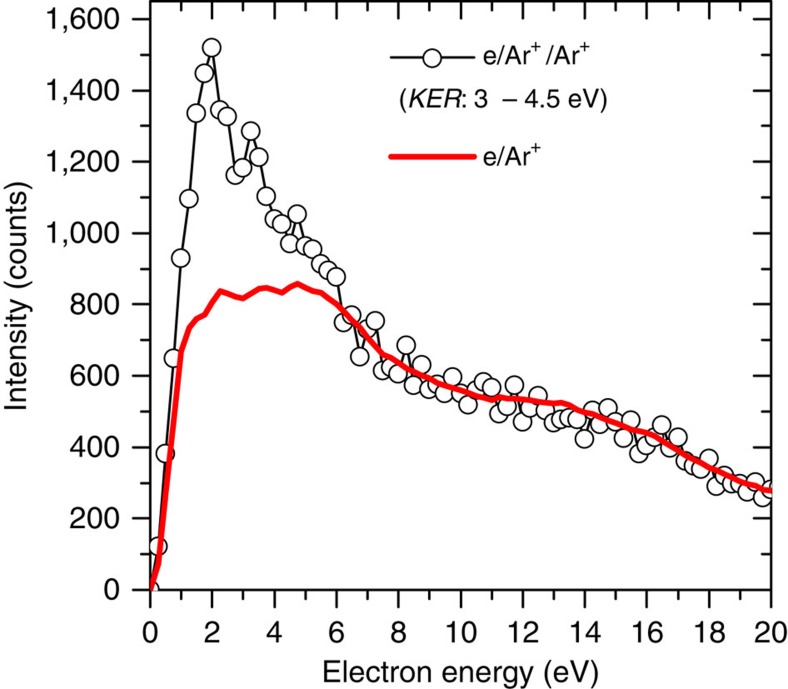
Measured electron energy distributions for *E*_0_=67 eV. Black open circles: the electron energy spectrum for ionization and fragmentation of argon dimers measured in e^−^/Ar^+^/Ar^+^ triple coincidences. The data are summed over the KER range from 3 to 4.5 eV . Red solid line: corresponding spectrum for ionization of argon monomers measured in e^−^/Ar^+^ double coincidences. The spectra were normalized to each other in the energy range from 10 to 15 eV.

**Figure 6:**
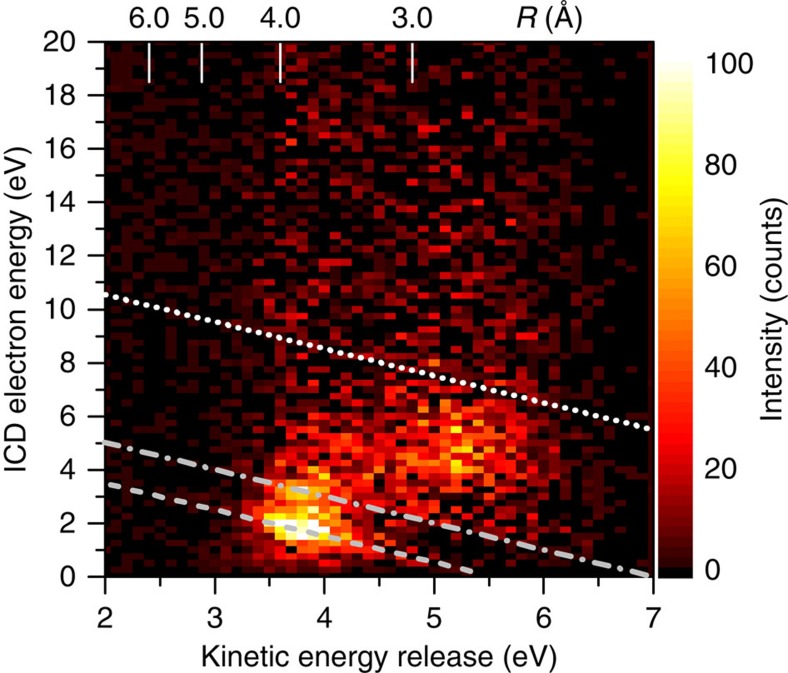
Measured two-dimensional energy map of electron and fragment ions for *E*_0_=67 eV. Correlation map between ICD electron energy (*E*_ICD_) and KER. The upper scale shows the internuclear distance *R* in Ångströms. Dashed line: position of ICD initial states Ar^+*^(3*p*^−2^(^1^D)4*p*^2^P,^2^F) and Ar^+*^(3*p*^−2^ (^1^D) 3*d*^2^P,^2^D). Dash–dotted line: position of states Ar^+*^(3*p*^−2^ (^1^D) 3*d*^2^S) and Ar^+*^(3*p*^−2^ (^3^P) 5*s*^2^P). Dotted line: maximum ICD electron energy for the Ar^+*^(3*p*^−2^ *nl*) series.

To pin down the ICD signature for the low-*E*_B_ region in Fig. 4 we identify the ICD electrons and show that their energy is correlated to the KER. In Fig. 5 the measured electron energy spectrum for the low-KER peak is shown (open circles). It contains a strong contribution from slow electrons produced in the initial ionization process (red line). It is obtained by the simultaneous recording of ionization of the Ar monomers in the target where ICD is absent and normalizing the resulting spectrum to the dimer spectrum in the energy range above 10 eV where no ICD electrons contribute (see Methods section). Clearly, an enhanced production of low-energy electrons (<8 eV) is observed in the electron energy spectrum of dimers compared with the result for monomers, which is the signature of ICD electron. The same procedure can be done for the high-KER electron spectrum (not shown here). If the monomer spectrum is subtracted from the total electron energy spectrum the pure ICD contribution correlated to the KER is obtained (Fig. 6). Here a clear correlation between ICD electron energy and ion KER is seen since for a particular ICD initial state Ar^+*^ with energy 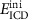 the relation must hold: 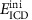
=2·IP_Ar_+*E*_ICD_+KER, where *E*_ICD_ is the ICD electron energy. Moreover low ICD electron energies are observed for the fast ICD process at KER=3.8 eV and higher ICD electron energies occur for slow process consistent with our discussion above. For the strongest line at *E*_ICD_≈1.8 eV 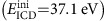 five terms of the two configurations Ar^+*^(3*p*^−2^ 4*p*) and Ar^+*^(3*p*^−2^ 3*d*) contribute. The second visible line at *E*_ICD_≈3.2 eV 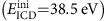 belongs to two terms of the configurations Ar^+*^(3*p*^−2^ 3*d*) and Ar^+*^(3*p*^−2^ 5*s*). For slow ICD channels a band of contributions is found at KER=5.2 eV and *E*_ICD_=3.0–6.0 eV, which can be assigned to a series of about 20 terms of the configurations Ar^+*^(3*p*^−2^ *nl*) with *nl*=4*d*, 4*f*, 5*d* and 6*s* (ref. 21).

### Separation of ICD and RCT contributions to the KER spectra

**Figure 7:**
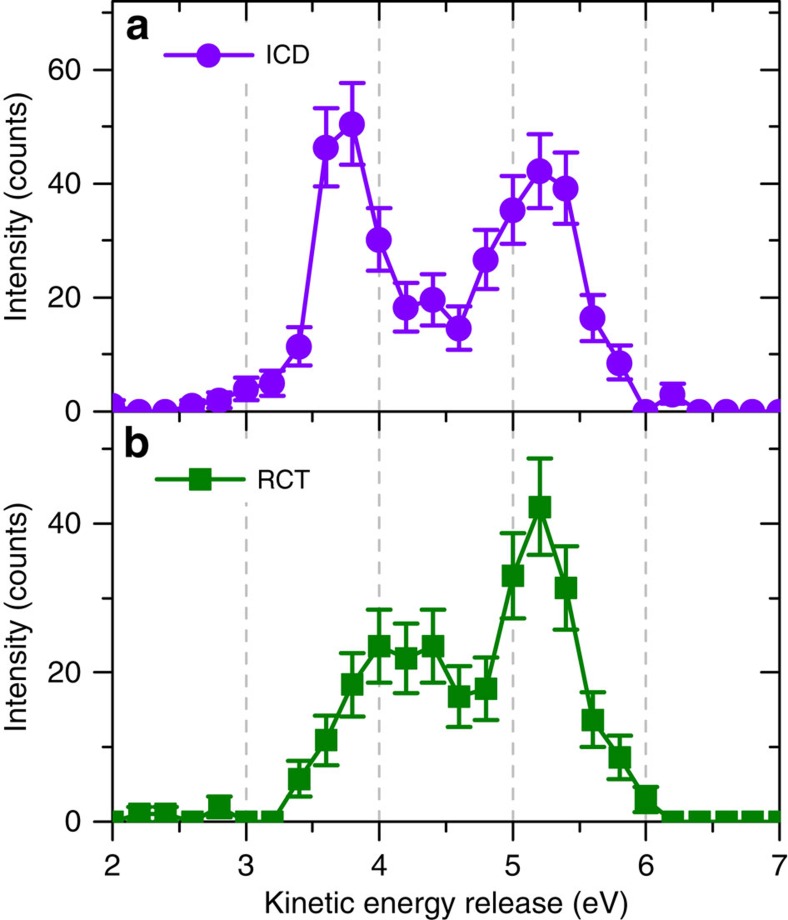
Measured KER distributions. The KER (**a**) for the ICD process and (**b**) for the RCT process. The error bars of the data points are defined as s.d. and are calculated as square root of the experimental count number.

Since we can separate ICD and RCT contributions, we may plot the KER spectrum for both relaxation mechanisms independently (Fig. 7). In ICD we observe two KER peaks at 3.8 and 5.2 eV, which agrees well with the calculations by Miteva *et al.*^18^ and the dynamics discussed above. The RCT yield exhibits a pronounced peak at 5.2 eV and a second peak (or an extended intensity) ranging down to 3.5 eV. This curve is consistent with simulated results for RCT obtained by Matsumoto *et al.*^22^ and is result of the long radiative lifetime of the doubly ionized state much longer than the vibrational period. The KER curve for RCT is the result of the weighted contributions of excited vibrational states and the *R*-dependent transition probability. In addition, we determine the relative importance of ICD to RCT at 67 eV to be 14 to 10.

### Role of sequential ionization

Finally, we consider direct Franck–Condon transitions to the repulsive Ar^+^−Ar^+^ state from the neutral dimer ground state, that is, the situation when the scattered electron consecutively kicks out one electron from each atom of the dimer. This process can give a contribution to the lower KER peak at 3.8 eV since it occurs at *R*_eq_ and it has identical binding energy as the ICD process. We do not find any signature for this process as, for example, a preferential production of Ar^+^−Ar^+^ pairs for an internuclear axis alignment along the projectile beam. From the cross-section for single ionization and the interatomic distance we estimate the probability for this process to be below 20% of the probability for ICD at *E*_0_=67 eV. Any possible contribution is eliminated largely from the electron spectrum in Fig. 5 by subtracting the direct single ionization part as discussed above.

## Discussion

Our work provides a detailed picture of the various relaxation mechanisms in small clusters utilizing full-coincidence measurements of all reaction products. We observe direct evidence of ICD and RCT in the decay of argon dimers ionized by electron impact. Recent years ICD processes were paid particular attention, since ICD is expected to be a general phenomenon in clusters and will be present also in hydrogen-bonding systems exposed to radiation, for example, liquid water^2^. It was proposed in refs 23, 24 that ICD is an abundant source for low-energy electrons in radiation damage of biological tissue^19^,^25^,^26^,^27^. Such low-energy electrons with energies below 10 eV are known to be partly responsible for the chemical transformation of DNA^15^,^16^ (in addition to the action of radicals), which leads to degradation of DNA and the starting point of cancer development. Since secondary electrons initially formed by ionizing radiation in cells have energies up to 100 eV (ref. 28), and drive many processes in aqueous systems^29^, electron-induced ICD processes as identified here provide the possibility to shift the energy distribution towards the more critical lower values^30^.

## Methods

### Experimental technique

The experiment was performed at the Max-Planck-Institut für Kernphysik in Heidelberg. A dedicated reaction microscope was used with two multichannel plate detectors with delay line position read-out for the coincident measurements of all reaction products (Fig. 1)^17^,^31^. In this apparatus a pulsed electron beam is crossed with an argon gas jet (2 mm diameter) containing a small fraction of about 2% dimers. The argon dimers were produced by supersonic expansion of pure argon gas through a 30-μm nozzle at room temperature and a typical stagnation pressure of 2 bar. The pulsed electron beam is emitted from an electron gun consisting of a tantalum photocathode, which is irradiated by ultraviolet-light pulses of 0.5 ns duration and electrostatic focusing lens elements. The energy width of the electron pulses is about 0.5 eV. The compact gun (8 mm × 50 mm=diameter × length) is placed in the centre of the ion drift region of the reaction microscope in a shielded housing such that it has negligible influence on the ion trajectories. An axial magnetic field (6.9 Gauss) guides the emitted collimated electron beam to the crossing zone with the gas jet and further to the beam dump, which is a central bore in the electron detector. Charged collisional products (electrons as well as ions) are extracted by means of a homogeneous electric field of 0.9 V cm^−1^ and projected onto two position- and time-sensitive multi-hit detectors. The guiding magnetic field also increases the transversal acceptance for electrons by restricting their transversal motion to cyclotron circles. The momentum vectors of the emitted species are determined by the impact positions on the detectors together with the corresponding times of flight. The acceptance angle for detection of electrons up to the energy of 15 eV is almost 4*π*, where small forward and backwards angles are excluded due to the presence of the beam dump in the electron detector. Ions originating from a Coulomb explosion have significantly higher momentum than the electrons observed, and require higher fields for efficient detection. Therefore, after 400 ns when the electrons have reached the detector the electric extraction field is ramped up to 20 V cm^−1^ for extraction of the ions. In this way full ion acceptance is reached up to a KER of 4 eV. For higher energies the decreasing ion acceptance is corrected for by scaling of the ion yield data with an energy dependent factor, which is obtained from simulating the spectrometer properties. The electron beam and spectrometer properties are calibrated using single ionization of atomic argon, which is the major constituent of the target gas jet. Here a binding energy resolution for the argon 3*p* orbital of about 2 eV is achieved. For the three electrons coincidence used to separate ICD and RCT a binding energy resolution of about 2.7 eV is estimated.

### Data reduction

In our experiment, monomer ionization is simultaneously recorded with dimer ionization and fragmentation. Since the ICD processes are absent for monomers, the monomer data can be considered as a reference to determine the continuous electron energy distribution due to scattered projectiles and ejected electrons from the ionizing collision. Thus, this contribution can be subtracted from the electron spectra recorded for the dimers to reveal the ICD electrons solely present for dimers. As one example the ionized electron energy spectra from monomer and dimer are presented in Fig. 5 in which the dimer spectrum is integrated over the KER range from 3 to 4.5 eV. The electron energy distributions agree well in the higher-energy region (>8 eV) because here the ICD process does not contribute^18^. Therefore, we normalized both spectra at the energy range from 10 to 15 eV. Comparing with the spectrum of monomer (e^−^/Ar^+^) the dimer result (e^−^/Ar^+^/Ar^+^) shows clear enhancement in the low-energy region (<8 eV) due to the presence of the ICD processes. Thus, the pure ICD electron spectrum is obtained and presented in Fig. 6 as a correlation diagram between electron energy and KER, where for each KER (±0.05 eV) the electron energy spectrum of monomer is normalized to the spectrum of dimer in the energy range from 10 to 15 eV to reduce the background. From the measured ICD electron energy (*E*_ICD_) and KER we can determine the binding energy of the ICD initial state as given below:


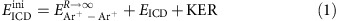


where 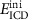 is the binding energy of the ICD initial state and 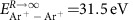 corresponds to the energy of the Ar^+^−Ar^+^ state at *R* close to infinity.

## Additional information

**How to cite this article:** Ren, X. *et al.* Direct evidence of two interatomic relaxation mechanisms in argon dimers ionized by electron impact. *Nat. Commun.* 7:11093 doi: 10.1038/ncomms11093 (2016).
